# Supplementary material for: Impact of histone deacetylase inhibition and arimoclomol on heat shock protein expression and disease biomarkers in primary culture models of familial ALS
Source: Cell Stress Chaperones. 2024 Apr 1;29(3):359–80. doi: 10.1016/j.cstres.2024.03.010 (PMC11015512; doi:10.1016/j.cstres.2024.03.010)
Supplement: Supplementary file 1 — Supplementary material [file mmc1.pdf]

Fernandez et al. Supplementary file

***Hspa1a* mRNA probes**
ttggttctgagtagctgtca, gctctgcttctcttgtcttc, tcgttggcgatgatctccac,
gtcgaacacggtgttctgcg, ttgtagttcacctgcacctt, ggaagaacgaccggctctcg, accatggacgagatctcctc,
agcgatctccttcatcttcg, gtgatcaccgcgttggtcac, cgctgagagtcgttgaagta, cacgtttagaccggcgatca,
cgtgggctcgttgatgatcc, gtcgaagatgagcacgttgc, tcgatcgtcaggatggacac, tggccttcacctcgaagatg,
tccacgaagtggctcaccag, ctggacgacagcgtcctctt, ctgagcacagctcttcgaac, atctgcgccttgtccatctt,
ttgaagaagtcctgcagcag, ttgatgctcttgttcaggtc,
tgcacgttctccgacttgtc, cttgatgagcgccgtcatca, ctcgtacacctggatcagca, ggatgccgctcagttcgaag,
gaaggtcacctcgatctgtg, tgacgttcaggatgccgttg, ctgagcttgcccttgagacc, cagcaccttcttcttgtcag,
gagatgacctcctggcactt,, cctggtacagcccactgatg, ctgagccagaggctcccttg, cagcagaggcctctaatcca
atatctgtctcctagccagc, ataatgacagtcctcaaggc, acttgattgcaggacaaact, gataaagcccacgtgcaata,
gacacacttgcagtgttaat, aaaacaaatcacatcagcgg, ggagagtccaaacacaaaga, atgtatcaacaatgtggccc,
ctctgaaggacccgacacaa, agtctccgctgtcagtaatc, taaacgcaaggagaagcagc, gtgcaaccaccatgcaagat,
caacctggaaacaagtccta, aaaactgaacacatgctggt, cggtaaaaattgacccgagt.

Hspa8 mRNA probes
Ttagacatggttgcttgtgt, gagatcaatgccaactgcag, ggcaataatttccacctttc, aaagcaacatagcttggcgt,
cattgcaacctgattcttgg, gatcagacgtttggcatcaa, cagcatcatcaaacctacgc, atcattcaccaccatgaagg,
tagaaactttttgtctcccc, agaaccatggaggacacttc, ctgcaatttccttcatcttt, atagttccagcatcttttg,
tcgaagtacattgaggccag ctccgaccttcttatctaag, gacacatcaaaagtgccacc, aatcttctccacctaagtgg,
atgattgaccattcggttgt, ctttcgcttgaactcagcaa,
cttgttctcactgatgtctt, tcaatctcaatactggcctg, gtcaattccctcatagagag,
cggaacaggtcagcattcaa, tcatggatctgtgacttgtc, gaatcttggggattctggta,
cagctcttttccattgaaga, aacagcttcatcggggttaa, cagacttgtctccagataga, agcagcaaatcctgaacgtt,
gggaaagaggagtgacatcc, gcttgatgaggacagtcatg, gtggtgaaagtctgtgtctg, tcaccttcatacacctgaat,
gtgagctcgaactttccaag, gtaacctcaatctgagggac, gcagaaacattgaggatgcc, tggtgatggtgatcttgttc,
gctcaatatcttccttactc, ttgtacttctcagcttcttg, gtgagttcttggaggaaacc, ttgctttcatgttgaaggca,
ttgccttgaagtttctcatc, agaatcttctgtttgtcctc, gctgatgatttcattgcact, ctctggtacagcttggtaat,
tgaagaagcaccaccagatg,
gacttaatccacctcttcaa, caaagctacaccttcttgga, tccatgttacttgttttggg

Supplementary Fig. 1. Sequence of Stellaris probes to mus musculus *Hspa1a* and *Hspa8* mRNA labeled with Quasar 570 or Quasar 670
S. Jacob-Tomas, et al., Using Single-Molecule Fluorescence Microscopy to Uncover Neuronal Vulnerability to Protein Damage, Methods Mol Biol. 2515 (2022) 237-254. 10.1007/978-1-0716-2409-8_15.

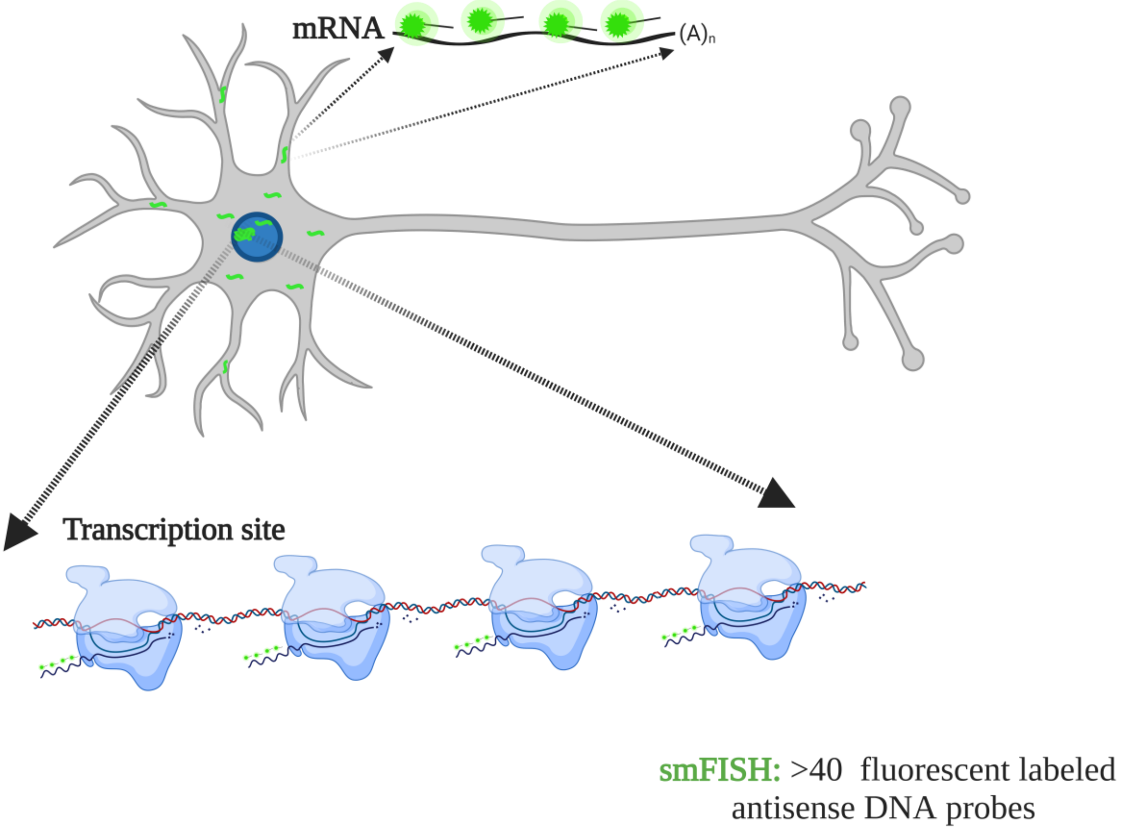

Supplementary Fig. 2. Single-molecule fluorescence *in situ* hybridization (smFISH) was used to investigate transcription sites within the cellular context. smFISH employs fluorescently labeled probes that specifically target RNA transcripts of interest. Forty individual antisense DNA probes to the same RNA molecules within the cell providing a robust smFISH signal for detection and imaging. Transcription sites are regions within the nucleus where active transcription is occurring, leading to the production of RNA molecules. The localization of RNA polymerase at transcription sites correlates with levels of mRNA transcription, resulting in brighter fluorescence signals compared to the smaller individual mRNA spots detected by smFISH. The depiction provided in Supplementary Figures 3 and 4 illustrates how transcription sites appear in an smFISH experiment. Within the nucleus of the cell, transcription sites typically manifest as big punctate fluorescent signals, representing the accumulation of RNA transcripts at the site of active transcription.

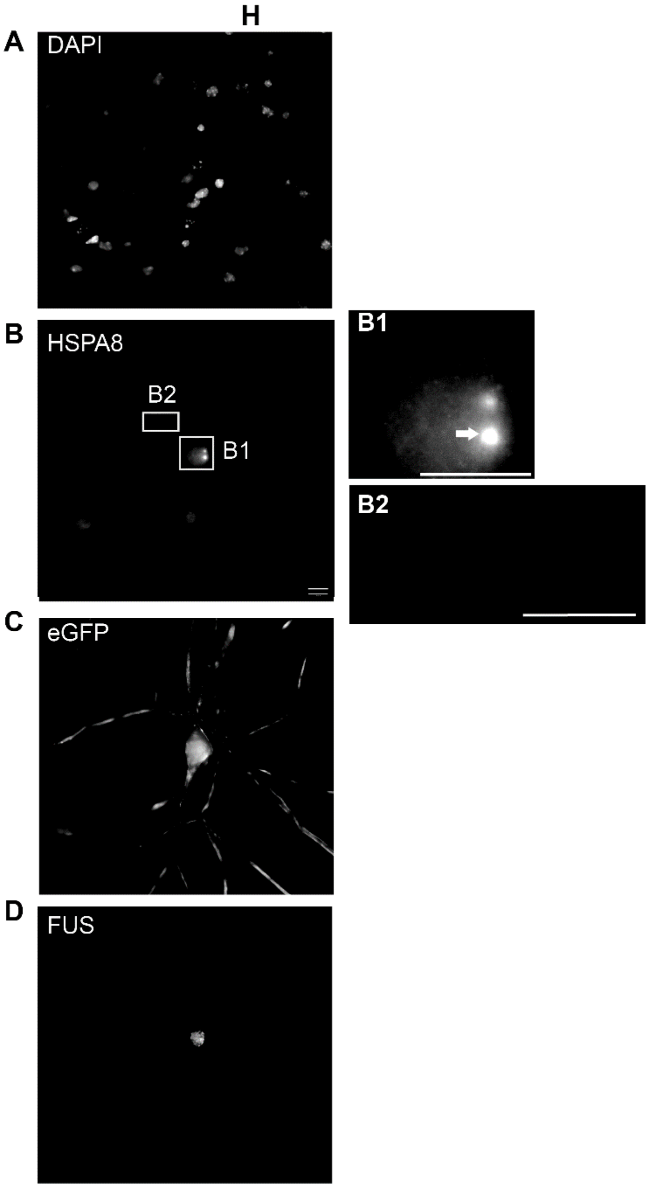

Supplementary Fig. 3. Representative neuron expressing eGFP and FUS^{R521G} showing images recorded from the various confocal channels. **A** DAPI channel. **B** smFISH for *Hspa1a* mRNA, both a wide-field view of the smFISH signal and zoomed-in images to showcase **B1** transcription sites and **B2** absence of *Hspa1a* mRNA spots in within dendrites. **C** The eGFP channel, which serves a dual purpose to facilitate the identification and visualization of microinjected neurons and to provide a reference marker for the pipeline software employed in our data analysis, facilitating automated analysis and quantification of experimental results. **D** FUS immunofluorescence. Scale bar = 10μm.

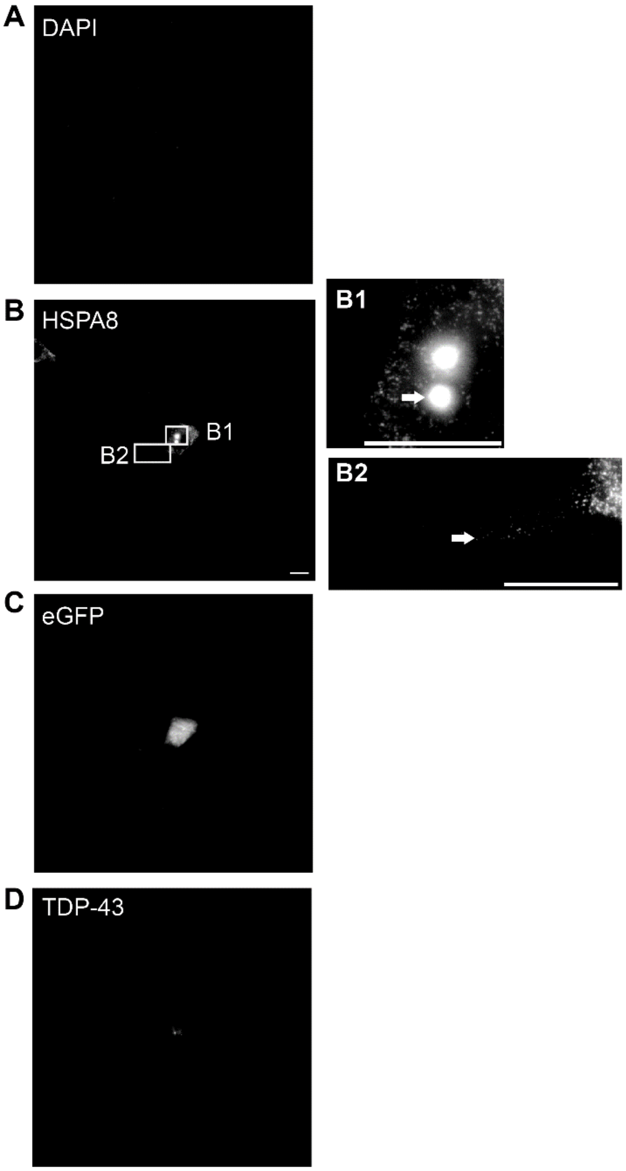

Supplementary Fig. 4. Representative neuron expressing eGFP and TDP-43^{G348C} showing images recorded from the various confocal channels. **A** DAPI channel. **B** smFISH for *Hspa8* mRNA, both a wide-field view of the smFISH signal and zoomed-in images to showcase **B1** transcription sites and **B2** *Hspa8* mRNA spots within the soma and dendrites (arrow). **C** The eGFP channel, which serves a dual purpose to facilitate the identification and visualization of microinjected neurons and to provide a reference marker for the pipeline software employed in our data analysis, facilitating automated analysis and quantification of experimental results. **D** FUS immunofluorescence. Scale bar = 10µm.

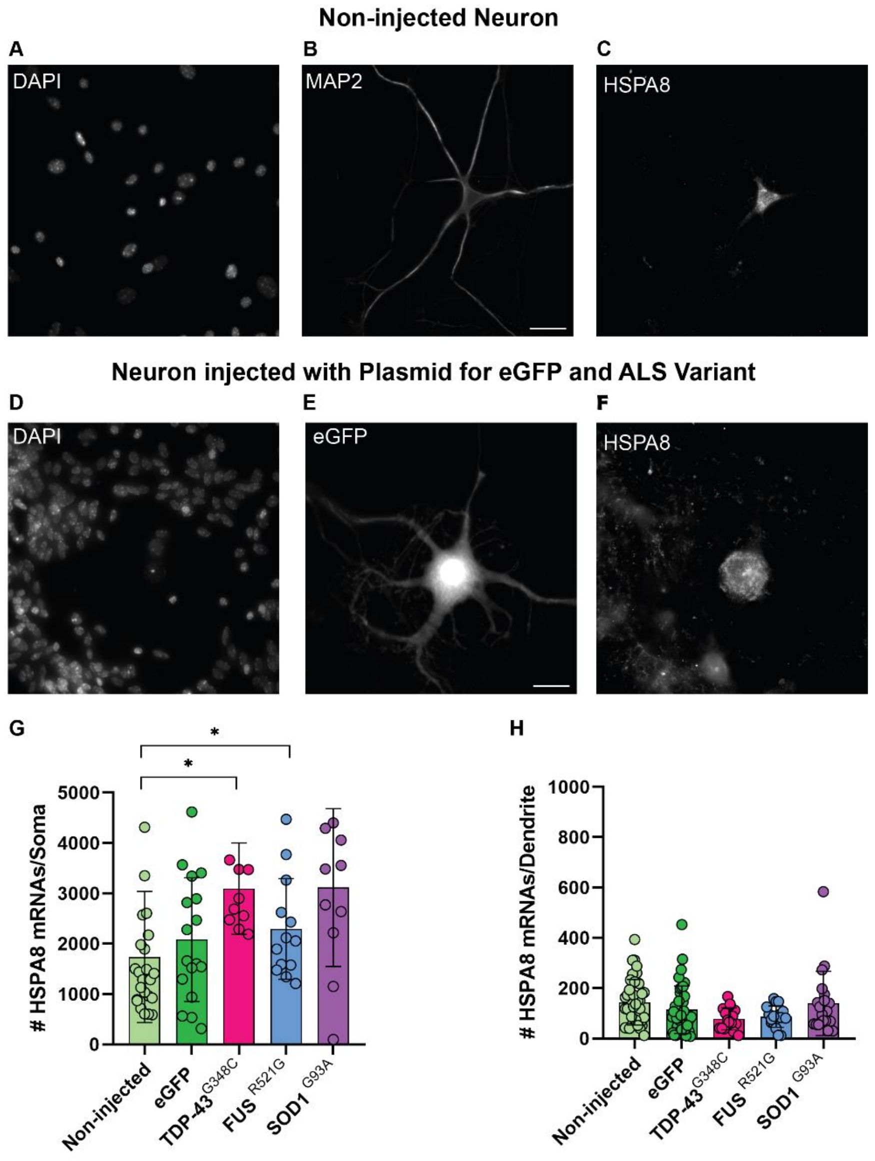

Supplementary Fig. 5. *Hspa8* mRNA levels in motor neurons after 24 hr, providing direct comparison of control (non-injected neurons and eGFP-expressing neurons) to neurons expressing ALS variants, complementing data presented in Fig. 5. Cultures were fixed and subjected to smFISH and immunocytochemistry. **A,B,C** Non-injected neuron identified by MAP2 labeling. **D**,**E**,**F** Neuron identified by eGFP epifluorescence. **G** *Hspa8* mRNA expression was significantly increased in somas of motor neurons expressing SOD1^{G93A}, and TDP-43^{G348C}. Expression of eGFP or FUS did not have a significant impact on the number of *Hspa8* mRNA. **H** No significant difference in *Hspa8* mRNA in dendrites. Data are presented as mean ± S.D., n = 9-60 neurons. Statistical significance was evaluated through one-way ANOVA followed by Bonferroni post hoc analysis. *$p<0.05$.

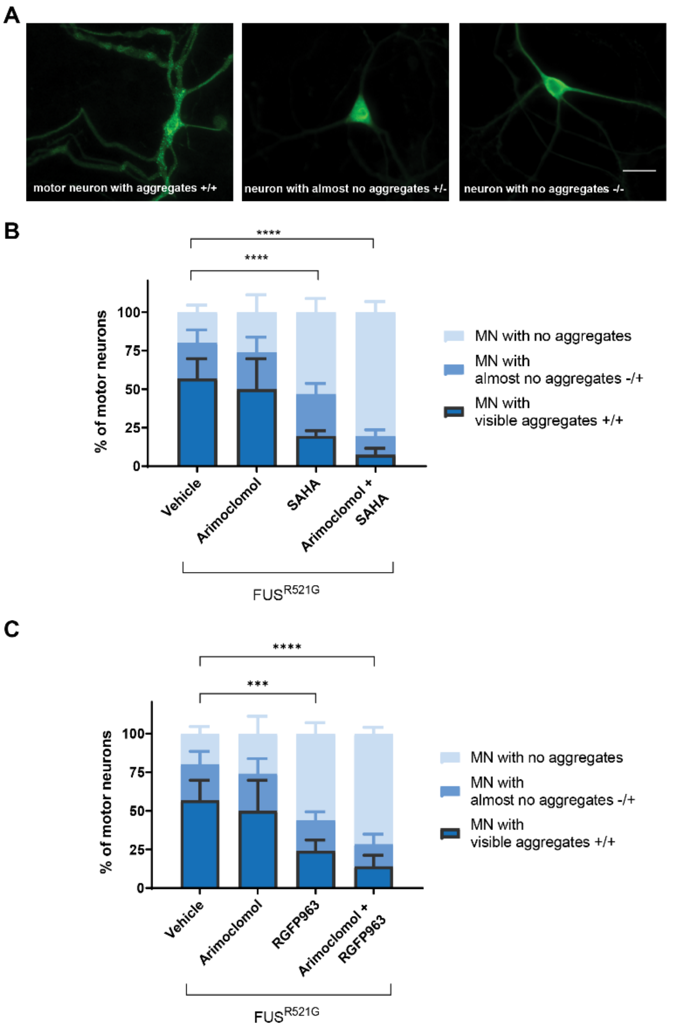

Supplementary Fig. 6. The impact of HDAC inhibition, arimoclomol, and combination treatment on aggregate formation in motor neurons expressing FUS^{R521}. FUS^{R521G} was expressed in motor neurons of dissociated spinal cord-DRG cultures by intranuclear microinjection of plasmid vector. Cultures were treated with vehicle (DMSO), 4 μM arimoclomol, or HDAC inhibitor (4μM SAHA or 1μM RGFP963) alone or in combination with arimoclomol for three days. Cultures were fixed and immunolabeled with FUS antibody. **A** The proportion of motor neurons exhibiting cytoplasmic aggregates was quantified as shown the micrographs: neurons with multiple aggregates throughout the cytoplasm (+/+), neurons with few aggregates (*/-), or neurons without visible aggregates (-/-). **B** SAHA and **C** RGFP963, alone and in combination with arimoclomol, but not arimoclomol alone, reduced the percentage of neurons with cytoplasmic aggregates. Data are presented as mean ± S.D. Each data point on the graph represents the mean % of neurons in each of 6-7 cultures (13-45 neurons/culture). Statistical significance was evaluated through one-way ANOVA followed by Bonferroni post hoc analysis. ***$p<0.001$ and ****$p<0.0001$. Scale bar = 20μm.

Supplementary Table 1. Summary of findings. → no significant change; ↑increase; ↓decrease; n.d. not determined. Included are findings from this study, Tradewell et al., 2011 [31], Tibshirani et al., 2015, 1017 [43,16] and Kuta et al., 2020 [9].

Parameter	Treatment	ALS Variant		
		TDP-43^{G348C}	FUS^{R521G}	SOD1^{G93A}
HSPA1A	Vehicle	↑	→	↑
	SAHA	↑	→	↑
	RGFP109	↑	→	↑
	RGFP963	↑	→	
	Tubastatin A	→	→	→
	Arimoclomol	→	→	↑
	Arimoclomol + HDAC inhibitor (SAHA, RGFP963 or RGFP109	↑	→	↑
Hspa1a mRNA	Vehicle	→	→	→
	RGFP963	→	→	↑
	Arimoclomol	→	→	↑
	RGFP963 + Arimoclomol	→	→	→
Hspa8 mRNA soma	Vehicle	↓	↓	↓
	RGFP963	→	→	↑
	Arimoclomol	→	→	→
	RGFP963 + Arimoclomol	↑	→	↑
Hspa8 mRNA dendrite	Vehicle	↓	↓	↓
	RGFP963	→	→	↑
	Arimoclomol	→	→	→
	RGFP963 + Arimoclomol	→	→	↑
Histone acetylation	Vehicle	↓	↓	n.d.
	SAHA	↑	↑	
	RGFP963	↑		
	Arimoclomol	→	→	
	SAHA + Arimoclomol	↑		
Nuclear TDP-43 or FUS	Vehicle	↓	↓	n.d.
	SAHA	↑	↑	
	RGFP109	↑	↑	
	RGFP963	↑		
	Tubastatin A	→	→	
	Arimoclomol	↑	↑	
	Arimoclomol + (SAHA, RGFP 109 or RGFP963	↑	↑	
Nuclear Brg1	Vehicle	↓	↓	n.d.
	SAHA	↑		
	RGFP963	↑		
	Arimoclomol	↑		
	RGFP963 + Arimoclomol	↑		
Mitochondrial transport	Vehicle	↓	↓	↓
	RGFP963	→		→
	Arimoclomol	→		→
	RGFP963 + Arimoclomol	→	→	↑
